# The learning curve of robotic esophagectomy: a systematic review with a proposed training and competency assessment framework

**DOI:** 10.3389/fonc.2026.1902156

**Published:** 2026-09-03

**Authors:** Konstantinos Kossenas, Adam Mylonakis, Dimitrios V. Avgerinos, Emmanouil I. Kapetanakis, Orestis Lyros, Ioannis Rouvelas, Ioannis Karavokyros, Dimitrios Schizas

**Affiliations:** 1 Second Propaedeutic Department of Surgery, National and Kapodistrian University of Athens, Laikon General Hospital, Athens, Greece; 2 First Department of Surgery, National and Kapodistrian University of Athens, Laikon General Hospital, Athens, Greece; 3 Third Cardiac Surgery Department, Onassis Cardiac Surgery Center, Athens, Greece; 4 Department of Thoracic Surgery, Red Cross General Hospital of Athens, Athens, Greece; 5 Fourth Department of Surgery, National and Kapodistrian University of Athens, Attikon University Hospital, Athens, Greece; 6 Division of Surgery and Oncology, Department of Clinical Science, Intervention and Technology (CLINTEC), Karolinska Institutet, Stockholm, Sweden; 7 Department of Upper Abdominal Diseases, Karolinska University Hospital, Huddinge, Stockholm, Sweden

**Keywords:** esophageal cancer, esophagectomy, learning curve, RAMIE, robot

## Abstract

**Background:**

Robotic-assisted minimally invasive esophagectomy (RAMIE) is a significant evolution in esophageal cancer surgery, providing improved visualization, dexterity and operative precision. However, RAMIE is technically demanding and has a steep learning curve. The goal of this systematic review was to assess the existing evidence on the learning curve of robotic esophagectomy and to develop a structured training and assessment framework for RAMIE.

**Methods:**

We performed a systematic literature search in PubMed/MEDLINE, Scopus and the Cochrane Library from inception to June 2026 according to the PRISMA 2020 statement. Studies that assessed the learning curve or implementation of robotic esophagectomy in adult patients were included. The outcomes included proficiency thresholds, operative time, recurrent laryngeal nerve (RLN) injury, complications, lymph node harvest, and factors affecting learning progression. The risk of bias was assessed with the Newcastle–Ottawa Scale (NOS).

**Results:**

We included 25 studies published between 2013 and 2026. Most studies were retrospective observational cohorts from high-volume centers in East Asia, Europe and North America. The most common assessment methodologies of the learning curve were based on cumulative sum (CUSUM) analysis. Typically, initial proficiency was attained after approximately 20–50 cases across studies, but stabilization of complications and optimization of oncologic quality often required higher procedural volumes. Increasing surgeon’s experience was associated with improvements in operative time, RLN injury, lymph node harvest, conversion rates, and postoperative complications. Prior experience in minimally invasive esophagectomy, structured proctoring pathways, high institutional volume, and team-based implementation models consistently enabled more rapid proficiency acquisition and safer RAMIE implementation.

**Conclusions:**

The learning curve for robotic esophagectomy is steep, but attainable. Structured training programs, dedicated robotic teams, and standardized implementation pathways may shorten the learning curve and enhance patient safety. Learning curve progression appears to be influenced by factors beyond case volume alone, supporting the development of structured training and assessment frameworks during RAMIE implementation. Based on the available evidence, we propose an evidence-informed expert framework that should be considered hypothesis-generating and requires prospective validation. Prospective multicenter studies and standardized reporting of learning curves are required to optimize training in robotic esophageal surgery and to establish universally accepted thresholds of proficiency.

**Systematic review registration:**

https://www.crd.york.ac.uk/PROSPERO/, identifier CRD420261417234

## Introduction

Esophageal cancer is still a major health problem worldwide. It is the seventh most commonly diagnosed cancer worldwide, with approximately 604,000 new cases each year, and the sixth leading cause of cancer-related death, with approximately 544,000 deaths annually (1), according to GLOBOCAN 2020 data. Geographic variation in the disease is marked, with the highest incidence in Eastern Asia and Eastern and Southern Africa, and the majority of global cases and deaths reported from Asia (1).

Surgical resection is the mainstay of curative treatment for localized esophageal cancer, and is often part of multimodal treatment strategies including neoadjuvant chemotherapy or chemoradiotherapy (2). Open esophagectomy (OE) has been the standard surgical approach historically, but in the past few decades, minimally invasive approaches have been embraced by a growing number of centers. Nevertheless, OE remains associated with considerable perioperative morbidity due to its invasive nature (3). Minimally invasive esophagectomy (MIE) has become increasingly established as an alternative to open surgery and may improve postoperative recovery while maintaining oncological outcomes (4).

More recently, RAMIE has emerged as a further iteration of minimally invasive esophageal surgery. The robotic platform offers several technical advantages, such as three-dimensional visualization, better ergonomics, tremor filtration, enhanced surgeon autonomy, and articulated instruments with increased dexterity, which can facilitate complex dissection and lymphadenectomy (5–9). Comparative studies have suggested that robotic esophagectomy may be an alternative to conventional minimally MIE, with the potential to further enhance surgical precision while maintaining perioperative and oncological outcomes (10, 11).

Despite these advantages, RAMIE remains one of the most technically demanding procedures in upper gastrointestinal surgery and has a significant learning curve (12). Several studies have attempted to define the learning curve using different methodologies including cumulative sum (CUSUM) analysis and sequential cohort comparison, but proficiency thresholds reported, differ significantly across studies. There is also significant heterogeneity in surgeon experience, institutional volume, operative technique and reporting of outcomes, which limits comparability and precludes standardized training recommendations (13).

Therefore, the aim of this systematic review was to perform a comprehensive assessment of the available evidence on the learning curve of robotic esophagectomy, to identify the factors influencing the learning process and to propose a standardized training and learning curve assessment framework for RAMIE.

## Methods

This study adhered to PRISMA 2020 and the Cochrane Handbook of Systematic Reviews (14, 15) and was prospectively registered with PROSPERO (ID: CRD420261417234).

### Population and PICO

This systematic review included adult patients who underwent robotic esophagectomy for benign or malignant esophageal diseases, including robotic-assisted minimally invasive esophagectomy (RAMIE), robotic McKeown esophagectomy, robotic Ivor Lewis esophagectomy, hybrid robotic esophagectomy, and total robotic esophagectomy. The intervention of interest was the implementation and learning curve of robotic esophageal surgery as evaluated by learning curve assessment methodologies such as cumulative sum (CUSUM), risk-adjusted CUSUM (RA-CUSUM), observed-expected CUSUM (O-E CUSUM), regression analysis, sequential cohort analysis, or comparative phase analysis. Early and late learning phases, stages of proficiency acquisition, robotic versus thoracoscopic minimally invasive esophagectomy learning curves and structured versus non-structured implementation pathways were compared. Outcomes of interest included cases needed to reach proficiency, operative time, recurrent laryngeal nerve (RLN) injury, complications, lymph node harvest, conversion rate, blood loss, length of stay, textbook outcomes, oncologic quality metrics, and factors impacting the learning curve including prior minimally invasive experience, institutional volume, structured training pathways, and team-based implementation models.

### Inclusion and exclusion criteria

Studies were included if they evaluated the learning curve, implementation, skill acquisition, or technical evolution of robotic esophagectomy in adult patients and reported quantifiable learning-related outcomes including cases to proficiency, operative time, complications, RLN injury, lymph node harvest, conversion rate, or perioperative outcomes. We included retrospective, prospective, comparative cohort, and institutional implementation studies of robotic esophagectomy techniques including RAMIE, robotic McKeown, robotic Ivor Lewis, hybrid robotic esophagectomy, and total robotic esophagectomy. We excluded reviews, editorials, commentaries, conference abstracts, letters, technical notes without outcome data, animal or cadaver studies, simulation-only studies, pediatric studies, and studies that did not specifically evaluate robotic esophagectomy learning curves. Studies were also excluded if they studied open or conventional minimally invasive esophagectomy without robotic surgery or did not report extractable learning curve data. Only studies published in English were considered in the present review. Dates of publication were not restricted during the selection of studies.

### Systematic search

A systematic literature search was performed in PubMed/MEDLINE, Scopus and the Cochrane Library from inception to June 1st, 2026. Search terms included combinations of “robotic esophagectomy”, “RAMIE”, “robot-assisted minimally invasive esophagectomy”, “Ivor Lewis”, “McKeown”, “learning curve”, “CUSUM”, “proficiency”, and “implementation” with Boolean operators. Reference lists of included studies were also screened for potentially eligible papers. Two independent reviewers manually removed duplicates (full search strategy available in **Supplementary File 1**).

### Data extraction

We extracted data on study characteristics, learning curve progression, and factors influencing acquisition of proficiency using pre-specified tables. Variables extracted included author, year, country, study design, sample size, type of robotic procedure, methodology used to assess the learning curve, and cases required to achieve proficiency. Other learning curve variables included early and proficiency phases, number of learning phases, primary proficiency metric, operative time, recurrent laryngeal nerve (RLN) injury, lymph node harvest, complications, and key study findings. Additionally, a novel extraction framework was developed to assess factors that may influence learning curve progression such as prior minimally invasive esophagectomy experience, structured training or proctoring pathways, institutional volume, and single-surgeon versus team-based implementation models.

### Study selection process

Two reviewers independently screened titles and abstracts from the literature search for potential eligibility. Full-text articles of potentially relevant studies were then independently evaluated against the predefined inclusion and exclusion criteria. Differences in study selection were resolved through discussion and consensus among reviewers.

### Definition of learning curve outcomes

Learning curve outcomes were categorized according to thresholds specific to each study, based on validated learning curve assessment methods such as CUSUM, RA-CUSUM, O-E CUSUM, regression analysis and sequential phase analysis. “Proficiency” was generally defined as the point at which operative performance, efficiency, or perioperative outcomes plateaued. “Stabilization” was defined as sustained improvement and consistency in surgical outcomes after the initial learning phase. “Optimization phase” was used to define later experience with further refinement of operative efficiency, complication reduction, and oncologic quality.

### Risk of bias assessment

The methodological quality of the included non-randomized studies was assessed using the Newcastle-Ottawa Scale (NOS) (16). The NOS evaluates studies across three major domains: selection of study groups, comparability of cohorts, and outcome assessment. Studies are awarded stars for each domain, with more stars denoting a lower risk of bias and higher methodological quality. Quality assessment was independently performed by two reviewers and any disagreement was settled by consensus.

The NOS showed overall moderate to high methodological quality, but it does not specifically assess the biases unique to surgical learning curve studies. There was considerable methodological heterogeneity regarding learning curve methodology including CUSUM implementation, use of RA-CUSUM or O-E CUSUM, primary endpoint selection, proficiency definitions, and thresholds used to define learning phases (20, 26, 28, 33, 35). Most studies were single-surgeon, retrospective cohorts without external validation, and thus results are subject to possible surgeon effects, institutional practice patterns, and arbitrary selection of inflection points. Therefore, the learning curve thresholds reported from around 20 cases up to more than 200 cases should be seen as context-dependent estimations, not as universally valid benchmarks.

## Results

### Search results

**Figure 1:**
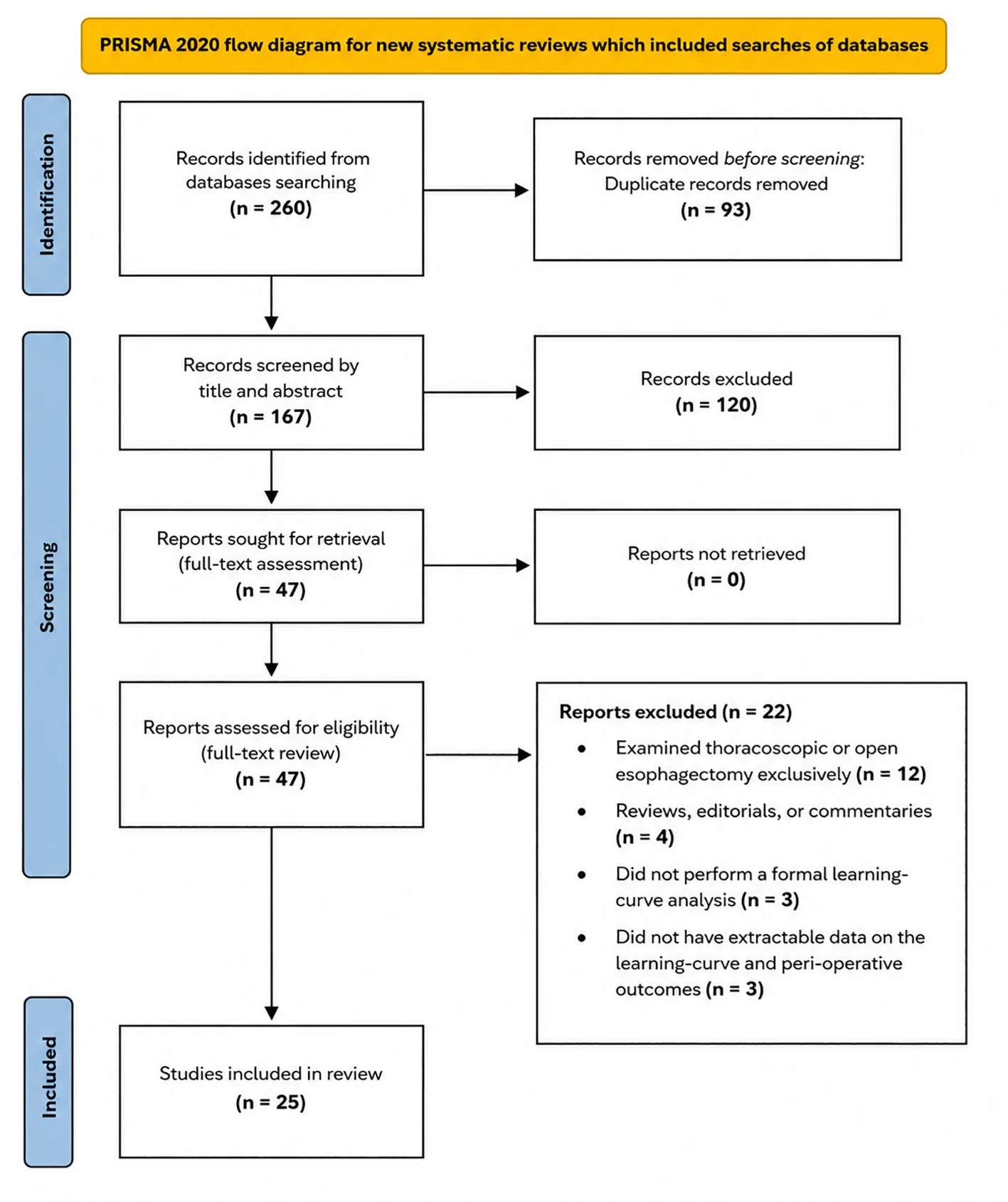
PRISMA 2020 flow diagram of study selection.

Database searches identified 260 studies. After removal of duplicates, 167 studies were screened by title and abstract and 47 studies were selected for full-text assessment. After full-text review, 22 studies were excluded. Twelve studies examined thoracoscopic or open esophagectomy exclusively, four were reviews, editorials, or commentaries, three did not perform a formal learning-curve analysis, and three did not have extractable data on learning-curve or peri-operative outcomes. Finally, 25 studies were included in the systematic review (**Figure 1**).

### Study characteristics and proficiency thresholds

In total, twenty-five studies were included, published between 2013 and 2026 (17–41). The majority of studies originated from East Asia, especially China, Japan and South Korea, which reflects the rapid adoption of robotic esophageal surgery in high-volume Asian centers (18, 20, 21, 25–33, 37, 39, 40). Further studies were performed in the USA (17, 19, 36), Europe (22, 24, 35, 38, 41), Taiwan (23) and Australia (34). The majority were retrospective observational studies, and a minority used prospective database designs (17, 24, 34). Detailed study characteristics and reported proficiency thresholds are summarized in **Table 1**.

**Table 1:** Study characteristics and proficiency thresholds.

Study	Country	Design	Patients (n)	Procedure	Learning curve method	Cases to proficiency
Hernandez et al. (2013) (17)	USA	Prospective database review	52	Robotic-assisted esophagogastrectomy	Sequential cohort comparison	20
Park SY et al. (2017) (18)	South Korea	Retrospective	33	Robotic esophagectomy + bilateral RLN dissection	Group comparison	20
Sarkaria et al. (2016) (19)	USA	Retrospective	100	RAMIE	Sequential cohort analysis	30–45
Park S et al. (2018) (20)	South Korea	Retrospective	140	RAMIE	O-E CUSUM	30–80 depending on metric
Zhang H et al. (2018) (21)	China	Retrospective	72	Robotic McKeown	CUSUM	26
van der Sluis et al. (2018) (22)	Netherlands	Retrospective comparative	312	Thoracoscopic RAMIE	CUSUM	70 (proctor), 24 (trained surgeon)
Chao et al. (2020) (23)	Taiwan	Retrospective	39 RE/67 VATE	Robotic esophagectomy	MA + CUSUM	12
Kingma et al. (2020) (24)	Germany	Prospective database review	70	RAMIE	CUSUM	22
Duan et al. (2020) (25)	China	Retrospective comparative	184	RAME vs VATE	CUSUM subgroup analysis	43
Yang et al. (2021) (26)	China	Retrospective	400	RAMIE-MK	CUSUM	40 learning; 80 quality
Zhuo et al. (2021) (27)	China	Retrospective	100	McKeown + Ivor Lewis RAMIE	CUSUM	23 McKeown +18 Ivor Lewis
Han et al. (2022) (28)	China	Retrospective	124	RAMIE Ivor Lewis	RA-CUSUM	51
Noshiro et al. (2022) (29)	Japan	Retrospective	81	Robotic McKeown	CUSUM	27
Hsieh et al. (2022) (30)	Taiwan/South Korea	Retrospective	179	McKeown RE	CUSUM	31/49
Sun et al. (2023) (31)	China	Retrospective	198 total (45 RAME)	RAME vs VATE	CUSUM	20
Jeon et al. (2023) (32)	South Korea	Retrospective	500	RAMIE	CUSUM	50 early; 150 stable
Duan et al. (2023) (33)	China	Retrospective	417	McKeown RAMIE	CUSUM	75/240
Narendra et al. (2023) (34)	Australia	Prospective database review	53 RAMIE	RAMIE	Regression analysis	22
Müller et al. (2023) (35)	Germany	Retrospective	154	RAMIE	CUSUM	55–83
Ramos-Fresnedo et al. (2025) (36)	USA	Retrospective	70	RAMIE Ivor Lewis	CUSUM	55
Yuan et al. (2025) (37)	China	Retrospective	83	RAMIE-MK	CUSUM	27
Hauge et al. (2025) (38)	Norway	Retrospective	200	RAMIE vs MIE	Comparative implementation analysis	~50
Sugase et al. (2026) (39)	Japan	Retrospective	50	Total RAMIE	CUSUM	18
Sato et al. (2026) (40)	Japan	Retrospective	30	Fusion surgery RAMIE	CUSUM	Progressive improvement
Jorek et al. (2026) (41)	Germany	Retrospective	71	Hybrid robotic Ivor Lewis	CUSUM	20/44

**Note:** Potential patient-cohort overlap may exist among studies originating from the same institutions or collaborative groups. In particular, Duan et al. (25) and Duan et al. (33) were conducted by the same Tianjin group and may include partially overlapping patients undergoing left recurrent laryngeal nerve lymph node dissection. Partial overlap may also exist among the Korean and Taiwanese recurrent laryngeal nerve cohorts reported by Park SY et al. (18), Chao et al. (23), and Hsieh et al. (30). Because the original studies evaluated different learning-curve outcomes and did not provide sufficient patient-level information, the extent of overlap could not be determined.

The initial experiences demonstrated comparatively short learning curves in early adoption of RAMIE. Early technical proficiency was reported to require approximately 20 cases by Hernandez et al. (17) and Park SY et al. (18). Sarkaria et al. (19) later proposed an even larger learning curve of 30–45 cases for robot-assisted minimally invasive esophagectomy (RAMIE) given the increasing complexity of the procedure and refinement of oncologic techniques.

Most of the recent studies applied cumulative sum (CUSUM)-based methods to assess learning curves (20–22, 24–33, 35–41). Proficiency thresholds differed significantly across studies based on operative approach, surgeon experience, institutional volume, and performance metrics selected. Robotic McKeown esophagectomy learning curve plateaued after 26–27 cases (21, 29, 37), but longer adaptation periods were described in larger institutional studies. Yang et al. (26) reported that about 40 cases were needed to overcome the initial learning curve and nearly 80 cases to reach a stable surgical quality. Similarly, Duan et al. (33) demonstrated a long multi-step learning process over 240 procedures.

The type of procedure also influenced learning time. Robotic Ivor Lewis esophagectomy had a lower learning curve than McKeown procedures (18 vs 23 cases), suggesting greater technical complexity of McKeown esophagectomy due to the cervical anastomosis and extensive upper mediastinal dissection (27). In contrast, Han et al. (28) reported a longer learning curve of 51 cases for robotic Ivor Lewis esophagectomy using risk-adjusted CUSUM analysis, underscoring the influence of complication-adjusted quality metrics.

Comparative studies also showed differences in implementation between the robotic and thoracoscopic approaches. Chao et al. (23) reported a relatively short robotic learning curve of 12 cases compared with video-assisted thoracoscopic esophagectomy (VATE), while Duan et al. (25) indicated that robotic-assisted minimally invasive esophagectomy required about 43 cases to reach stable proficiency.

Structured implementation pathways appeared to significantly reduce the learning curve. van der Sluis et al. (22) showed that surgeons with a formal robotic training program became proficient after 24 cases compared to an estimated 70 cases for the initial proctor. Similarly, Kingma et al. (24) demonstrated the safe implementation of RAMIE after a median of 22 procedures using a standardized training pathway.

Institutional studies of large volumes indicated that procedural mastery extends beyond initial technical adaptation. Jeon et al. (32) reported an initial learning curve of about 50 cases and stable long-term results after nearly 150 procedures. Müller et al. (35) also reported different thresholds of proficiency ranging from 38 to 83 cases depending on the outcome parameter being evaluated. More recent studies suggest that accumulated institutional experience and technological evolution may shorten learning curves with Sugase et al. (39) reporting proficiency after only 18 cases during total RAMIE implementation.

Generally, most studies suggested that initial proficiency in robotic esophagectomy requires about 20–50 cases, while much higher volumes may be required to optimize oncologic quality, complication rates and long-term outcomes (20, 22, 26, 32, 33, 35). However, the substantial variability in reported proficiency thresholds highlights the influence of procedural complexity, surgeon background, training pathways, and institutional experience on learning curve progression.

### Patterns of proficiency acquisition in robotic esophagectomy

The learning curve for robotic esophagectomy seems to be a dynamic and multi-phased process with gradual improvements in operative efficiency, complication reduction, oncologic quality, and technical standardization. The learning phases, outcome measures, and key findings of the included studies are summarized in **Table 2**.

**Table 2:** Learning curve characteristics and key findings.

Study	Early phase	Proficiency phase	Number of phases	Main metric used	Key finding
Hernandez et al. (2013) (17)	First 20 cases	After 20 cases	2	Operative time	Proficiency achieved after 20 robotic esophagogastrectomies
Park SY et al. (2017) (18)	Initial 20 cases	Later 13 cases	2	Vocal cord palsy	RLN palsy significantly decreased after 20 cases
Sarkaria et al. (2016) (19)	First 30–45 cases	After 45 cases	2	Operative time	Standardized RAMIE and dedicated team improved safety and efficiency
Park S et al. (2018) (20)	1–30/1–60/1–80	After each threshold	Multiple	O-E CUSUM	Different perioperative outcomes improved at different experience thresholds
Zhang H et al. (2018) (21)	1–26	27–72	2	Total surgical time	26 cases required for early proficiency
van der Sluis et al. (2018) (22)	Proctor: 1–70; trainee: 1–24	After thresholds	2	OT, blood loss, conversion	Structured proctoring dramatically shortened learning curve
Chao et al. (2020) (23)	1–12	>12	2	RLN palsy (CUSUM)	Prior VATE experience shortened robotic RLN learning curve
Kingma et al. (2020) (24)	1–22	23–70	2	Thoracic OT and blood loss	Structured training pathway enabled safe implementation
Duan et al. (2020) (25)	1–43	44–70	2	Left RLN LN harvest	Improved RLN dissection and lower RLN injury after proficiency
Yang et al. (2021) (26)	1–40	Phase II: 41–215 plateau; Phase III: 216–400 descending/optimized	3	CUSUM	40 cases for learning phase; quality outcomes improved after 80 procedures
Zhuo et al. (2021) (27)	McKeown: 1–23; Ivor Lewis transition: next 18	After thresholds	4	Thoracic LN yield	Transition from McKeown to Ivor Lewis created second learning phase
Han et al. (2022) (28)	1–51	52–124	2	RA-CUSUM major complications	51 cases required for technical proficiency
Noshiro et al. (2022) (29)	1–27	>27	2	CUSUM + ERA utilization	Standardized use of extra robotic arm accelerated learning
Hsieh et al. (2022) (30)	Surgeon A: 1–31; Surgeon B: 1–15 and 16–49	After thresholds	3	RLN palsy	Prior thoracoscopic experience strongly affected learning curve
Sun et al. (2023) (31)	1–20	>20	2	Upper mediastinal LN harvest	Early proficiency achieved after 20 cases
Jeon et al. (2023) (32)	1–50 learning; 51–150 developing	151–500 stable	3	CUSUM	50 cases needed for proficiency and >150 for stabilization
Duan et al. (2023) (33)	1–75	Phase II: 76–240; Phase III: 241–404	3	RLN LN harvest	RLN injury and complications decreased progressively
Narendra et al. (2023) (34)	1–22	>22	2	Regression of OT	RAMIE learning curve shorter than previous MIE experience
Müller et al. (2023) (35)	1–55	38–83 depending on parameter	Multiple	Multi-dimensional CUSUM	Learning curve varies according to performance parameter
Ramos-Fresnedo et al. (2025) (36)	1–55	56–70	2	Operative time	Technical proficiency acquired after 55 cases
Yuan et al. (2025) (37)	1–27	28–83	2	Total operative time	27 cases required for RAMIE-MK proficiency
Hauge et al. (2025) (38)	First 50 RAMIE cases	Last 50 RAMIE cases	2	Operative outcomes	Outcomes improved during later implementation phase
Sugase et al. (2026) (39)	Phase I: 1–6; Phase II: 7–18	Phase III: 19–50	3	Abdominal OT	Abdominal proficiency achieved after ~18 cases
Sato et al. (2026) (40)	Early da Vinci phase	Continued hinotori phase	Progressive	Console operation time	Skills transferred smoothly across robotic platforms
Jorek et al. (2026) (41)	1–20; complications until 44	>44	2	CUSUM complications/textbook outcome	Textbook outcomes achievable during learning curve

Early studies mainly reported on operative time and perioperative outcomes. Hernandez et al. (17) described significant improvements in operative efficiency after the first 20 robotic esophagogastrectomies, and Park SY et al. (18) found a reduction in vocal cord palsy after the first 20 procedures. Sarkaria et al. (19) also considered the first 30–45 cases the main adaptation phase and stressed the importance of procedure standardization and specialized robotic teams.

A number of studies have shown that the learning curves for robotic esophagectomy are more complicated than the traditional two-phase model. Park et al. (20) reported several inflection points at approximately 30, 60 and 80 cases using observed-expected CUSUM analysis. Yang et al. (26) described a three-phase progression with an initial learning phase, plateau phase and final optimization phase beyond 200 procedures. Similar results were also found by Jeon et al. (32) and Duan et al. (33) who demonstrated improvements in recurrent laryngeal nerve (RLN) injury, postoperative complications, and operative consistency that persisted well beyond early technical proficiency. Importantly, learning curves varied depending on the chosen outcome parameter. Müller et al. (35) showed that there were different cut-offs of proficiency for operative time, complications and other perioperative outcomes suggesting that robotic esophageal surgery is multidimensional.

Moreover, several studies focused only on RLN dissection, which is one of the most technically challenging parts of robotic esophagectomy. Chao et al. (23) showed that substantial prior experience with thoracoscopy can decrease the RLN learning curve. Duan et al. (25) reported improved RLN lymph node retrieval and lower RLN injury rates after about 43 cases. Hsieh et al. (30) also demonstrated significant inter-surgeon variability with the learning time significantly affected by the prior thoracoscopic experience.

Technical standardization also seemed to make learning smoother. As demonstrated by Noshiro et al. (29), the regular use of an additional robotic arm improved exposure and accelerated adaptation in robotic McKeown esophagectomy. Similarly, Sun et al. (31) reported early proficiency in upper mediastinal lymph node dissection after about 20 cases.

Recent studies have highlighted the changing influence of technological innovation and procedural transition. Zhuo et al. (27) showed that changing from McKeown RAMIE to robotic Ivor Lewis esophagectomy created a second learning curve despite previous robotic experience. Sugase et al. (39) reported a rapid acquisition of the abdominal phase during total RAMIE implementation, while Sato et al. (40) demonstrated progressive improvements during the transition between robotic platforms, suggesting transferability of robotic surgical skills.

Subsequent studies more frequently assessed advanced quality metrics rather than operative efficiency alone. Han et al. (28) found stabilization of major complications after approximately 51 cases using risk-adjusted CUSUM analysis whereas Ramos-Fresnedo et al. (36) achieved proficiency after 55 robotic Ivor Lewis procedures. Jorek et al. (41) also showed that despite persistently elevated complication rates during early implementation, textbook outcomes can still be achieved during the learning curve.

Overall, the available evidence suggests that robotic esophagectomy proficiency develops through multiple overlapping phases, with different performance metrics.

### Factors influencing the learning curve

Several factors were consistently associated with the length and development of the robotic esophagectomy learning curve, including prior experience in minimally invasive esophagectomy (MIE), structured training pathways, institutional volume and team-based implementation models. An overview of factors influencing learning curve progression is presented in **Table 3**.

**Table 3:** Factors influencing learning curve progression.

Study	Prior MIE experience	Proctoring/structured training	High-volume center	Single surgeon vs team
Hernandez et al. (2013) (17)	Yes – proficient in minimally invasive esophagogastrectomy	No formal program reported	No	Single surgeon
Park SY et al. (2017) (18)	Not clearly specified	No	No	Single surgeon
Sarkaria et al. (2016) (19)	Yes	Yes – standardized RAMIE technique and dedicated OR team	Yes	Dedicated 2-surgeon team
Park S et al. (2018) (20)	Yes	No formal program reported	Yes	Single surgeon
Zhang H et al. (2018) (21)	Yes – open and thoracolaparoscopic experience	No	Yes	Single surgical team
van der Sluis et al. (2018) (22)	Yes	Yes – structured proctoring pathway	Yes	Proctor + trainee surgeon
Chao et al. (2020) (23)	Yes – extensive VATE experience	No	No	Single surgeon
Kingma et al. (2020) (24)	Yes – prior conventional MIE	Yes – tailored structured pathway with proctoring	Yes	Single surgeon
Duan et al. (2020) (25)	Yes	No	Yes	Single surgical group
Yang et al. (2021) (26)	Not clearly specified	No formal program described	Yes	Single surgeon
Zhuo et al. (2021) (27)	Yes – experienced esophageal team	No	Yes	Single surgical team
Han et al. (2022) (28)	Not clearly specified	No	No	Single surgeon
Noshiro et al. (2022) (29)	Not clearly specified	Yes – standardized extra robotic arm utilization	No	Single surgeon
Hsieh et al. (2022) (30)	Compared extensive vs limited VATE experience	Informal prior robotic assisting	Yes	Two surgeons
Sun et al. (2023) (31)	Yes – extensive VATE experience	No	Yes	Single surgical team
Jeon et al. (2023) (32)	Not clearly specified	No	Yes	Institutional experience
Duan et al. (2023) (33)	Not clearly specified	No	Yes	Institutional surgical team
Narendra et al. (2023) (34)	Yes – prior MIE experience	Careful implementation strategy	Yes	Single surgeon
Müller et al. (2023) (35)	Yes – experienced RAMIE surgeons	Yes – proficiency-based progression pathway	Yes	Two experienced surgeons
Ramos-Fresnedo et al. (2025) (36)	Not clearly specified	No	Yes – tertiary referral center	Institutional team
Yuan et al. (2025) (37)	Yes – open and thoracolaparoscopic esophagectomy + animal robotic training	Yes – systematic robotic training	No	Single surgeon
Hauge et al. (2025) (38)	Yes – prior MIE program	Institutional implementation pathway	Yes	Institutional team
Sugase et al. (2026) (39)	Yes – experienced RAMIE center	Yes – two-team simultaneous approach	Yes	Team-based approach
Sato et al. (2026) (40)	Prior da Vinci RAMIE experience	Fusion Surgery concept	No	Surgical team
Jorek et al. (2026) (41)	Yes – experienced high-volume center	Yes – structured robotic training program	Yes	Defined surgical team

Previous thoracoscopic or MIE experience was one of the strongest predictors of accelerated proficiency (17, 19–21, 25, 27, 31, 34, 35, 37–39, 41). Chao et al. (23) and Hsieh et al. (30) demonstrated that previous thoracoscopic expertise significantly reduced the learning curve of RLN dissection. Yuan et al. (37) further stressed the importance of multimodal preparation including open, thoracolaparoscopic and animal robotic training prior to clinical implementation.

Formal proctoring pathways and structured training also significantly shortened learning duration. Sarkaria et al. (19) emphasized the need for a standardized RAMIE technique and a dedicated operating room team. van der Sluis et al. (22) concluded that surgeons trained via a formal robotic pathway became proficient significantly faster than the original proctor surgeon. Similar findings have been reported by Kingma et al. (24), where safe implementation was demonstrated after approximately 22 procedures with a tailored structured training program.

Institutional surgical volume was another major driver of proficiency. Most studies with favorable outcomes and shorter learning curves were published from high-volume esophageal surgery centers (19–22, 24–27, 30–35, 38, 39, 41). High-volume programs are likely to benefit from standardized perioperative pathways, dedicated multidisciplinary support, and continuous technical refinement. Jeon et al. (32), Duan et al. (33) and Hauge et al. (38) all stressed the importance of institutional experience to achieve stable long-term results.

The way robotic programs are organized has also changed over time. Previous studies have described single-surgeon learning models (17, 18, 20, 23, 26, 28, 29, 34, 37), but more recent studies have more often focused on team-based and institutional implementation strategies (19, 21, 22, 24, 25, 27, 31–33, 35, 36, 38–41). Dedicated team structures were important in complex robotic esophagectomy programs in particular, allowing knowledge transfer, optimizing workflows, and standardizing procedures.

Taken together, the available evidence suggests that learning curve progression is influenced not only by individual surgeon experience, but also by structured training, institutional expertise, and the broader organizational framework in which robotic esophagectomy is implemented.

### Risk of bias assessment

The methodological quality of included studies was overall high to moderate. The detailed Newcastle–Ottawa Scale assessment is presented in **Table 4**.

**Table 4:** Newcastle–Ottawa Scale (NOS) assessment of included studies.

Study	Selection (4)	Comparability (2)	Outcome (3)	Total score	Risk of bias
Hernandez et al. (2013) (17)	★★★	★	★★	6	Moderate
Park SY et al. (2017) (18)	★★★	★	★★	6	Moderate
Sarkaria et al. (2016) (19)	★★★★	★★	★★★	9	Low
Park S et al. (2018) (20)	★★★★	★★	★★★	9	Low
Zhang H et al. (2018) (21)	★★★	★	★★	6	Moderate
van der Sluis et al. (2018) (22)	★★★★	★★	★★★	9	Low
Chao et al. (2020) (23)	★★★	★	★★	6	Moderate
Kingma et al. (2020) (24)	★★★★	★★	★★★	9	Low
Duan et al. (2020) (25)	★★★★	★★	★★★	9	Low
Yang et al. (2021) (26)	★★★★	★★	★★★	9	Low
Zhuo et al. (2021) (27)	★★★	★	★★	6	Moderate
Han et al. (2022) (28)	★★★	★	★★	6	Moderate
Noshiro et al. (2022) (29)	★★★	★	★★	6	Moderate
Hsieh et al. (2022) (30)	★★★★	★★	★★★	9	Low
Sun et al. (2023) (31)	★★★	★	★★	6	Moderate
Jeon et al. (2023) (32)	★★★★	★★	★★★	9	Low
Duan et al. (2023) (33)	★★★★	★★	★★★	9	Low
Narendra et al. (2023) (34)	★★★★	★	★★★	8	Low
Müller et al. (2023) (35)	★★★★	★★	★★★	9	Low
Ramos-Fresnedo et al. (2025) (36)	★★★	★	★★	6	Moderate
Yuan et al. (2025) (37)	★★★★	★★	★★★	9	Low
Hauge et al. (2025) (38)	★★★★	★★	★★★	9	Low
Sugase et al. (2026) (39)	★★★	★	★★	6	Moderate
Sato et al. (2026) (40)	★★★	★	★★	6	Moderate
Jorek et al. (2026) (41)	★★★★	★★	★★★	9	Low

★ = 1 point; ★★ = 2 points; ★★★ = 3 points; ★★★★ = 4 points.

The majority of studies were retrospective observational cohorts from high-volume centers with well-defined patient populations and standardized pathways for robotic esophagectomy. Several studies had high NOS scores because of structured implementation protocols and appropriate outcome assessment, and adequate follow-up. Nevertheless, a moderate risk of bias remained across several studies due to retrospective single-center study designs, lack of adjustment for comparators, absence of blinded or structured outcome assessment, and inadequate reporting of selection methodology and control for confounding factors. No randomized controlled trials were identified. Overall, the findings of this review should therefore be interpreted in the context of predominantly retrospective evidence derived from high-volume institutions with varying learning curve assessment methodologies.

## Discussion

This systematic review showed that robotic esophagectomy has a steep but achievable learning curve with most studies showing proficiency after around 20 to 80 cases, depending on the outcome measured and the analytical method used. Improvement in operative time, lymph node yield, conversion rates, RLN injury and post-operative complications was associated with increasing surgeon experience. High-volume centers, prior experience with minimally invasive esophagectomy, structured training pathways, and dedicated robotic teams seemed to promote more rapid proficiency and safer implementation of RAMIE.

The crucial issue is how the RAMIE learning curve compares with the traditional learning curve for minimally invasive esophagectomy. Thoracoscopic dissection, particularly near the recurrent laryngeal nerves and upper mediastinum, and thoracoscopic anastomosis may be technically more challenging because of rigid instrumentation, limited articulation and less optimal ergonomics. The robotic platform can overcome some of these technical limitations through 3D visualization, tremor filtration and articulated instruments. However, the present review demonstrates that prior experience with conventional minimally invasive esophagectomy is still a significant facilitator of RAMIE proficiency (17, 19–21, 23, 25, 27, 30, 31, 34, 35, 37–39, 41). Thus, robotic technology does not substitute for advanced esophageal surgical experience, but may enable surgeons with established thoracoscopic and minimally invasive skills to translate their knowledge of anatomy, operative sequencing, complication avoidance, and team coordination to the robotic platform. Conversely, surgeons with previous robotic experience from less complex procedures may learn console control and instrument handling more quickly, but still need to acquire procedure-specific expertise in esophageal mobilization, lymphadenectomy, conduit preparation, anastomosis and perioperative management. Thus, the learning curve for RAMIE should be viewed as a function of robotic platform experience and previous complex esophageal surgical experience, rather than an isolated case-volume threshold.

Previous reviews have attempted to systematically evaluate the learning curve of RAMIE. The present review offers a more targeted and up-to-date review of the robotic esophagectomy learning curve in RAMIE as compared to the systematic review by Prasad et al. (13) which combined thoracoscopic, hybrid, and minimally invasive esophagectomy, rather than a single, homogeneous procedure-based review. Prasad et al. (13) predominantly reviewed generic MIE learning curves, and reported wide ranges of proficiency with operative time as the main benchmark. Our review incorporated more contemporary high-volume robotic studies with advanced methodologies such as O-E CUSUM, RA-CUSUM and multi-phase learning models. Our review also examined procedure-specific factors related to RLN dissection, changes in technology between robotic platforms, structured training pathways, and textbook outcomes, thereby providing a more nuanced understanding of the multidimensional and evolving nature of proficiency in robotic esophagectomy. In general, our results were in line with those of Prasad et al. (13) confirming that the learning curve for robotic esophagectomy is highly variable and dependent on the chosen outcome measures. Our review however included more recent RAMIE-specific studies and demonstrated that proficiency is often a dynamic multi-phase process beyond operative efficiency alone and incorporates RLN outcomes, textbook outcomes and technical standardization. Furthermore, our results were largely consistent with those of Pickering et al. (12). Both studies demonstrated considerable variability in RAMIE learning curve lengths and highlighted the importance of structured training and proctorship during adoption. Our review also included newer studies and provided a more comprehensive assessment of multi-phase learning models, RLN-specific learning curves, technological transitions, and advanced quality metrics such as textbook outcomes and risk-adjusted complication analyses, highlighting the evolving and multidimensional nature of proficiency in robotic esophagectomy.

A major strength of this review is the comprehensive assessment of learning curve outcomes from multiple institutions, countries and robotic techniques and the investigation of factors affecting proficiency such as prior experience, proctoring and center volume. The review additionally focused on clinically meaningful perioperative and oncologic outcomes rather than operative time alone. However, most of the included studies were retrospective and from high-volume centers which could introduce selection bias and limit generalizability. There was considerable heterogeneity in definitions of learning curve, outcome reporting and analytical methods, which precluded quantitative meta-analysis. Additionally, many of the studies were single-surgeon experiences with limited long-term oncologic follow-up. Therefore, the reported proficiency thresholds should be interpreted with caution and may not be directly transferable across institutions or training environments. Several studies were conducted by the same high-volume institutions, notably the Tianjin group in China and Korean/Taiwanese collaborative centers, which may indicate some patient overlap between cohorts studying different aspects of the RAMIE learning curve, especially RLN dissection (18, 23, 25, 30, 33). Although these studies addressed different research questions and outcome measures, the possibility of duplicate inclusion of overlapping patients cannot be completely excluded and may have disproportionately influenced the qualitative synthesis. The search strategy is also a methodological limitation in the review. Only studies published in English were included, which may have introduced language bias. In addition, Embase was not searched. Most of the included studies were from East Asia, thus excluding Embase may have led to ignoring relevant regional publications not indexed in PubMed or Scopus. Therefore, the evidence available may not be representative of the global experience of RAMIE implementation. These limitations are reflective of the current state of the evidence in robotic surgery generally. Across many surgical specialities, the literature is mainly retrospective observational studies from high-volume expert centers, with variable reporting of learning curves, patient selection criteria, surgical techniques and outcomes, thus limiting the external validity and generalizability of reported proficiency thresholds (42–49).

**Figure 2:**
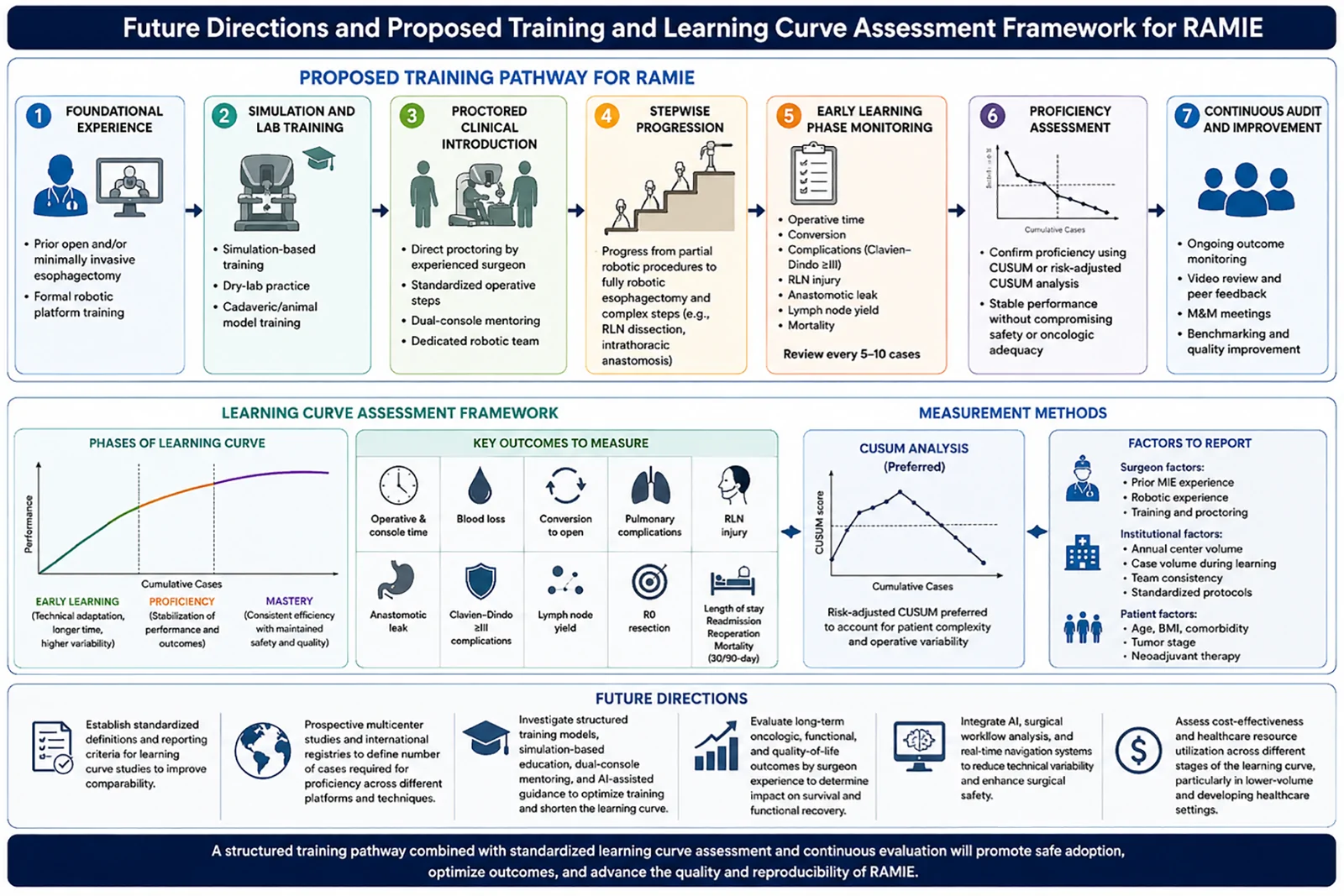
Proposed evidence-informed expert framework for training and learning curve assessment in robot-assisted minimally invasive esophagectomy (RAMIE). The framework is conceptual and requires prospective validation.

Importantly, the available evidence suggests that proficiency in robotic esophagectomy cannot be defined by case numbers alone. Rather, learning curve progression appears to be influenced by a combination of surgeon experience, procedural complexity, institutional volume, structured training pathways, and team-based implementation strategies. These observations formed the basis for the proposed training and learning curve assessment framework presented in **Figure 2**.

This framework is an expert proposal based on evidence and narrative synthesis of the available literature rather than a formally developed consensus statement or validated competency model. It was not developed using formal consensus methodology (eg, Delphi) and has not been prospectively clinically validated. It should therefore be considered hypothesis-generating and requires prospective multicenter validation before widespread implementation.

The authors believe, based on their broader complex minimally invasive and robotic surgery, that a staged approach to implementation is more appropriate for complex robotic procedures than a direct progression to a fully robotic operation. Practical measures include previous experience in open and minimally invasive esophageal surgery, simulation and dry-laboratory training, observation of established robotic programs, standardization of procedures, proctoring during the initial cases, and use of a consistent multidisciplinary operating-room team. The selection of early cases should lean towards technically suitable patients, with more complex cases being added only when operative steps and team workflows are reproducible. Regular audit of operative time, conversion, recurrent laryngeal nerve injury, anastomotic complications, lymph-node yield and other quality outcomes may help to identify areas for further training. These observations are the experienced reflections of the authors based on their experience and should not be taken as independently validated evidence.

As robotic upper gastrointestinal surgery is gaining ground (50–57), future studies should aim to develop standardized definitions and reporting guidelines for robotic esophagectomy learning curves to improve comparability between studies. A structured training pathway may enable safe implementation of robot-assisted minimally invasive esophagectomy (RAMIE). Surgeons should have ideally prior experience with open and/or minimally invasive esophagectomy, followed by robotic simulation, dry-lab, cadaveric training and proctored clinical introduction with standardized operative steps and dedicated robotic teams. A step-wise approach from partial robotic procedures to full robotic esophagectomy may further optimize patient safety and skill acquisition.

Further prospective multicenter studies and international registries are needed to better define proficiency thresholds across robotic platforms and operative techniques. Learning curve assessment should encompass different phases of learning: early learning, proficiency and mastery. Evaluation should include clinically meaningful perioperative and oncologic outcomes such as complications, recurrent laryngeal nerve injury, anastomotic leak, lymph node yield, conversion, R0 resection and mortality. Use of CUSUM and risk-adjusted CUSUM analysis seems to be among the most robust methods currently available for determining proficiency thresholds.

Future studies should also examine structured training models, dual console mentoring, simulation-based education, and artificial intelligence-assisted surgical guidance to optimize robotic training pathways and minimize technical variability. The long-term outcomes for oncologic, functional, quality of life and cost effectiveness based on surgeon experience should also be evaluated in future research.

## Conclusion

The results suggest that robotic esophagectomy can be performed safely in experienced centers and within the context of structured training programs. Standardized mentorship, proctoring pathways, and dedicated robotic teams might be of help in decreasing the learning curve and improving patient safety. Previous experience with minimally invasive esophagectomy also appears to facilitate robotic skill acquisition. These findings may align with the centralization of complex robotic esophageal surgery to high-volume centers with the requisite expertise and infrastructure.

## Data Availability

The original contributions presented in the study are included in the article/**Supplementary Material**. Further inquiries can be directed to the corresponding author.
