# Supplementary material for: The learning curve of robotic esophagectomy: a systematic review with a proposed training and competency assessment framework
Source: Front Oncol. 2026 Sep 3;16:1902156. doi: 10.3389/fonc.2026.1902156 (PMC13581900; doi:10.3389/fonc.2026.1902156)
Supplement: Supplementary file 1 [file Supplementaryfile1.docx]

| S1: Search strategy | | |
| --- | --- | --- |
| Database | Search | Results |
| PubMed | (("Esophagectomy"[Mesh] OR esophagectom*[tiab] OR oesophagectom*[tiab] OR "esophageal resection"[tiab] OR "oesophageal resection"[tiab])  AND  ("Robotic Surgical Procedures"[Mesh] OR robotic[tiab] OR robot-assisted[tiab] OR robotically[tiab] OR "robot assisted"[tiab] OR RAMIE[tiab] OR RAE[tiab])  AND  ("Learning Curve"[Mesh] OR "learning curve"[tiab] OR "learning curves"[tiab] OR proficiency[tiab] OR competency[tiab] OR "operative experience"[tiab] OR "surgical experience"[tiab] OR "case volume"[tiab] OR "cumulative sum"[tiab] OR CUSUM[tiab] OR LC-CUSUM[tiab] OR "risk-adjusted CUSUM"[tiab] OR "RA-CUSUM"[tiab])) | 110 |
| Scopus | TITLE-ABS-KEY ( ( esophagectom* OR oesophagectom* OR "esophageal resection" OR "oesophageal resection" ) AND ( robotic OR "robot-assisted" OR "robot assisted" OR robotically OR RAMIE OR RAE ) AND ( "learning curve" OR "learning curves" OR proficiency OR competency OR "operative experience" OR "surgical experience" OR "case volume" OR "cumulative sum" OR CUSUM OR "LC-CUSUM" OR "risk-adjusted CUSUM" OR "RA-CUSUM" ) ) | 147 |
| Cochrane Library | (  (esophagectom* OR oesophagectom* OR "esophageal resection" OR "oesophageal resection")  AND  (robotic OR "robot-assisted" OR "robot assisted" OR robotically OR RAMIE OR RAE)  AND  ("learning curve" OR "learning curves" OR proficiency OR competency OR "operative experience" OR "surgical experience" OR "case volume" OR "cumulative sum" OR CUSUM OR "LC-CUSUM" OR "risk-adjusted CUSUM" OR "RA-CUSUM")  ) | 3 |
